# Endoscopic management of pancreatic and biliary duct stenoses due to a giant pseudoaneurysm in a patient clinically suggestive of Loeys-Dietz syndrome

**DOI:** 10.1007/s12328-025-02151-w

**Published:** 2025-05-28

**Authors:** Tomohiro Tanikawa, Akihisa Akagi, Mayuko Kawada, Katsunori Ishii, Takashi Fushimi, Noriyo Urata, Mitsuhiko Suehiro, Hidenori Shiraha, Ken Haruma, Hirofumi Kawamoto

**Affiliations:** 1 Department of General Internal Medicine 2, Kawasaki Medical School, 2-6-1, Nakasange, Kita-Ku, Okayama, 700-8505 Japan; 2 Department of General Surgery, Kawasaki Medical School, 2-6-1, Nakasange, Kita-Ku, Okayama, 700-8505 Japan

**Keywords:** Loeys–Dietz syndrome, Pseudoaneurysm, Pancreaticobiliary obstruction, Transcatheter arterial embolization, Endoscopic drainage

## Abstract

Loeys–Dietz syndrome is a rare connective tissue disorder characterized by the formation of aggressive arterial aneurysms. There are a few reports of Loeys–Dietz syndrome with pseudoaneurysms causing simultaneous pancreatic and biliary stenoses. Herein, we report the case of a 42-year-old man with Loeys–Dietz syndrome who presented with acute pancreatitis and liver dysfunction caused by a giant pancreaticoduodenal artery pseudoaneurysm compressing the main pancreatic and bile ducts. To minimize the risk of pseudoaneurysm rupture during endoscopic intervention, transcatheter arterial embolization was performed, followed by endoscopic intervention. Although initial clinical improvement was observed after endoscopic stent placement, a fistula between the pancreatic duct and the thrombosed pseudoaneurysm was detected at 4 months but spontaneously closed with continued stenting. Despite persistent ductal stenosis requiring long-term stent management, the fistula had closed 1 year after the initial stent placement. To the best of our knowledge, this is the first report describing a structured treatment strategy for pancreaticobiliary obstruction caused by a Loeys–Dietz syndrome-related pseudoaneurysm. This case highlights the importance of a stepwise interventional radiology-first approach and careful follow-up for the management of complex vascular compressive syndromes.

## Introduction

Loeys–Dietz syndrome (LDS) is a rare, systemic hereditary connective tissue disorder caused by mutations in transforming growth factor beta receptor 1 (TGFBR1) or TGFBR2. These mutations lead to dysregulated transforming growth factor-β (TGF-β) signaling, vascular fragility, and aggressive aneurysm formation [1, 2]. Patients with LDS often experience arterial dissection and aneurysms in various arteries, most commonly involving the aorta [3]. Visceral artery aneurysms not only pose a risk of critical rupture but can also cause organ dysfunction by compressing surrounding structures, potentially leading to complications such as ischemia or obstruction, including the common bile duct (CBD) and main pancreatic duct (MPD). Aneurysms have the potential to cause bile duct obstruction and obstructive pancreatitis. Previous reports have described cases of bile duct stenosis or obstructive jaundice due to various aneurysms [4, 5]. However, there are few reports of obstructive pancreatitis due to aneurysms.

Herein, we present a case report of a patient with LDS with a giant pancreaticoduodenal artery pseudoaneurysm leading to simultaneous obstructive pancreatic and biliary duct stenosis. This case was successfully managed via a stepwise approach using transcatheter arterial embolization (TAE) followed by endoscopic biliary and pancreatic drainage. To the best of our knowledge, this is the first report outlining a management strategy for pancreatic and bile duct stenosis caused by an LDS-associated pseudoaneurysm.

## Care report

**Fig. 1:**
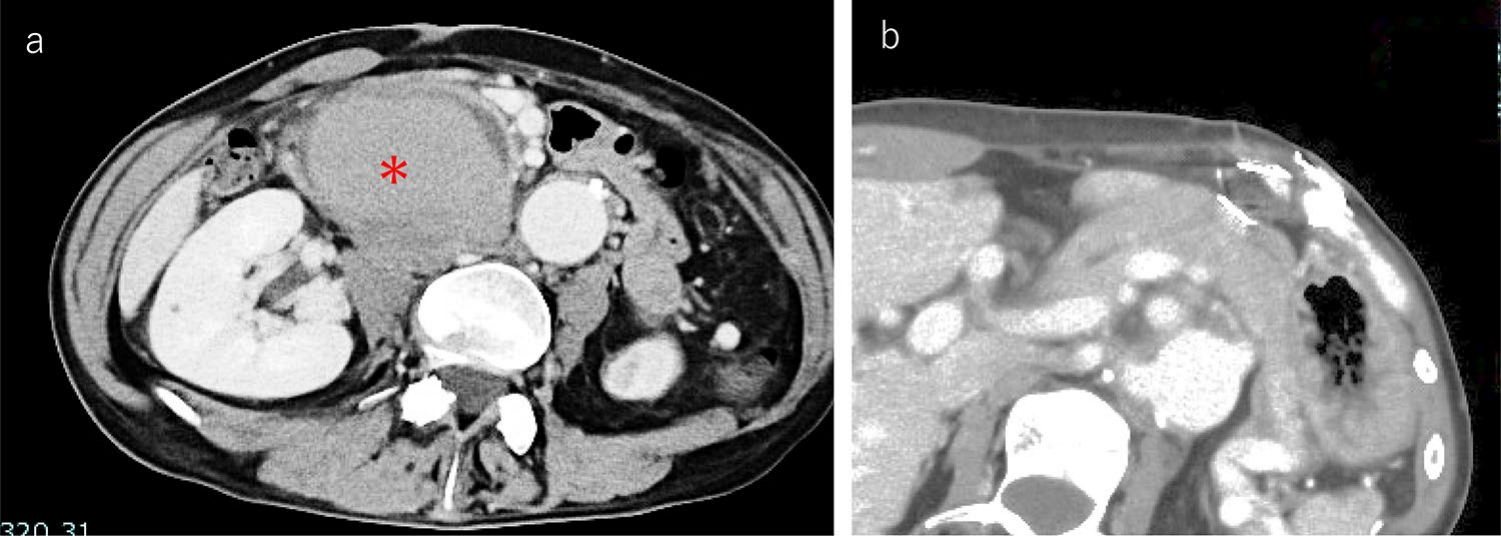
Computed tomography at the delayed phase showing a pseudoaneurysm compressing the pancreatic head and bile duct. **a** CT revealed a 70 mm pseudoaneurysm (*) arising from the posterior superior pancreaticoduodenal artery, which exhibited delayed contrast enhancement, indicating residual blood flow. And it compressed the pancreatic head. **b** CT showed slight dilation of the main pancreatic duct and pancreatic swelling

A 42-year-old man with a clinical diagnosis of Loeys–Dietz syndrome (LDS) was referred to our hospital for worsening epigastric and back pain. This patient had been followed up and treated at a previous institution as a patient with suspected LDS based on multiple systemic aneurysms and clinical features. Genetic testing revealed a heterozygous variant in TGFBR2 (c.1219A > C, p.Thr407Pro, NM_003242.6), currently classified as a variant of uncertain significance (VUS). Although a definitive genetic diagnosis had not been established, no mutations were detected in genes associated with Marfan syndrome or vascular Ehlers–Danlos syndrome, and the overall clinical picture was highly suggestive of LDS. He had undergone multiple interventional radiology procedures for arterial complications, including aortic dissection (Stanford A), left internal carotid artery aneurysm, and bronchial artery aneurysm. Gradually, the intra-abdominal pseudoaneurysm increased in size, and while discussing the treatment plan, he developed epigastric pain and back pain. Blood examination revealed elevated pancreatic enzyme levels (amylase: 3,677 mg/dL, lipase: 6,373 U/L) and liver enzyme levels (AST: 313 U/L, ALT: 162 U/L, ALP: 333 U/L, γ-GTP: 118 U/L). Contrast-enhanced computed tomography (CT) identified a 70-mm pseudoaneurysm arising from the posterior superior pancreaticoduodenal artery, compressing the pancreatic head. This resulted in slight dilation of the MPD in the pancreatic body and tail, along with pancreatic swelling, indicative of acute pancreatitis (Fig. 1). In addition, the pseudoaneurysm compressed the CBD, causing CBD stenosis and liver dysfunction. Based on these findings, the patient was diagnosed with pseudoaneurysm-induced pancreatic and biliary obstructions. Endoscopic biliary and pancreatic drainage were necessary to manage these complications. However, endoscopic intervention carried a risk of pseudoaneurysm rupture due to endoscope and device manipulation. To avoid this risk, TAE was performed before endoscopic therapy.

**Fig. 2:**
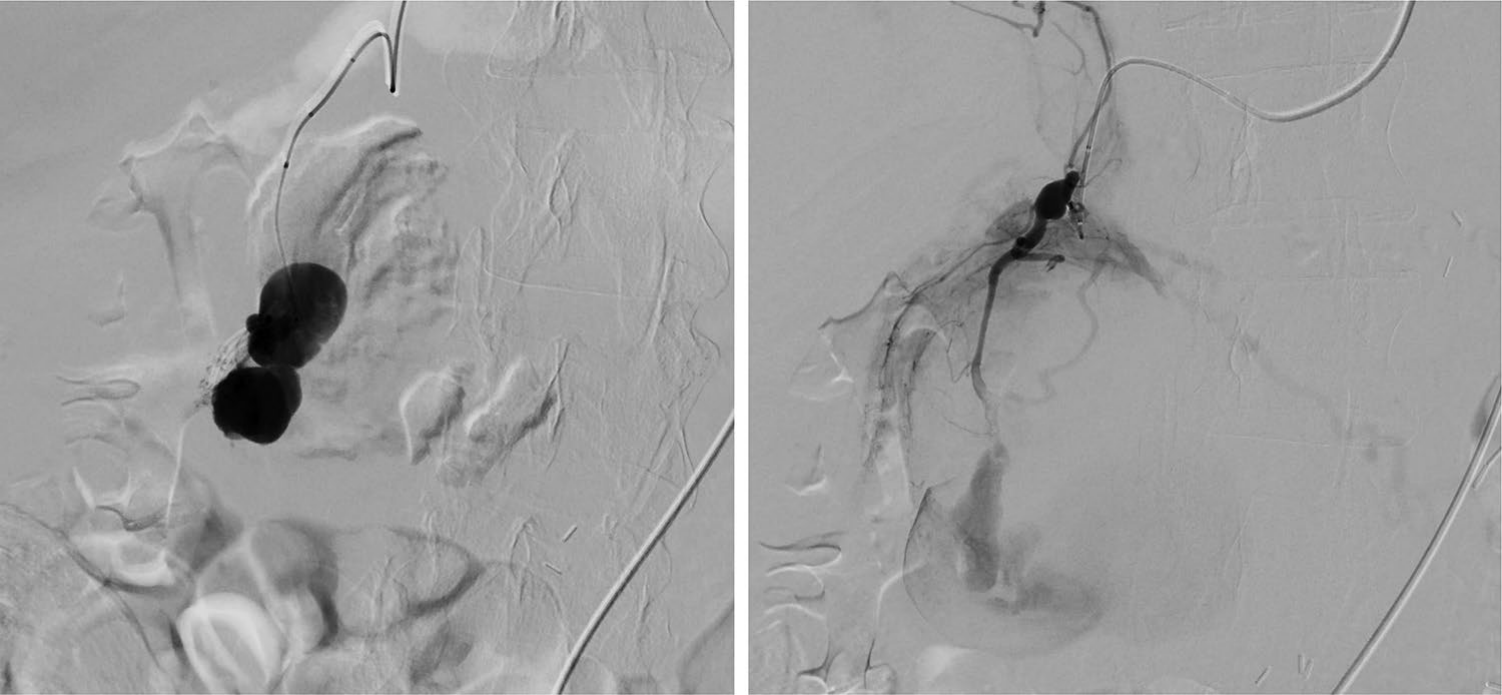
Transcatheter arterial embolization (TAE). TAE was performed first using n-butyl-2-cyanoacrylate and coils, successfully occluding the aneurysm from the posterior superior pancreaticoduodenal artery

**Fig. 3:**
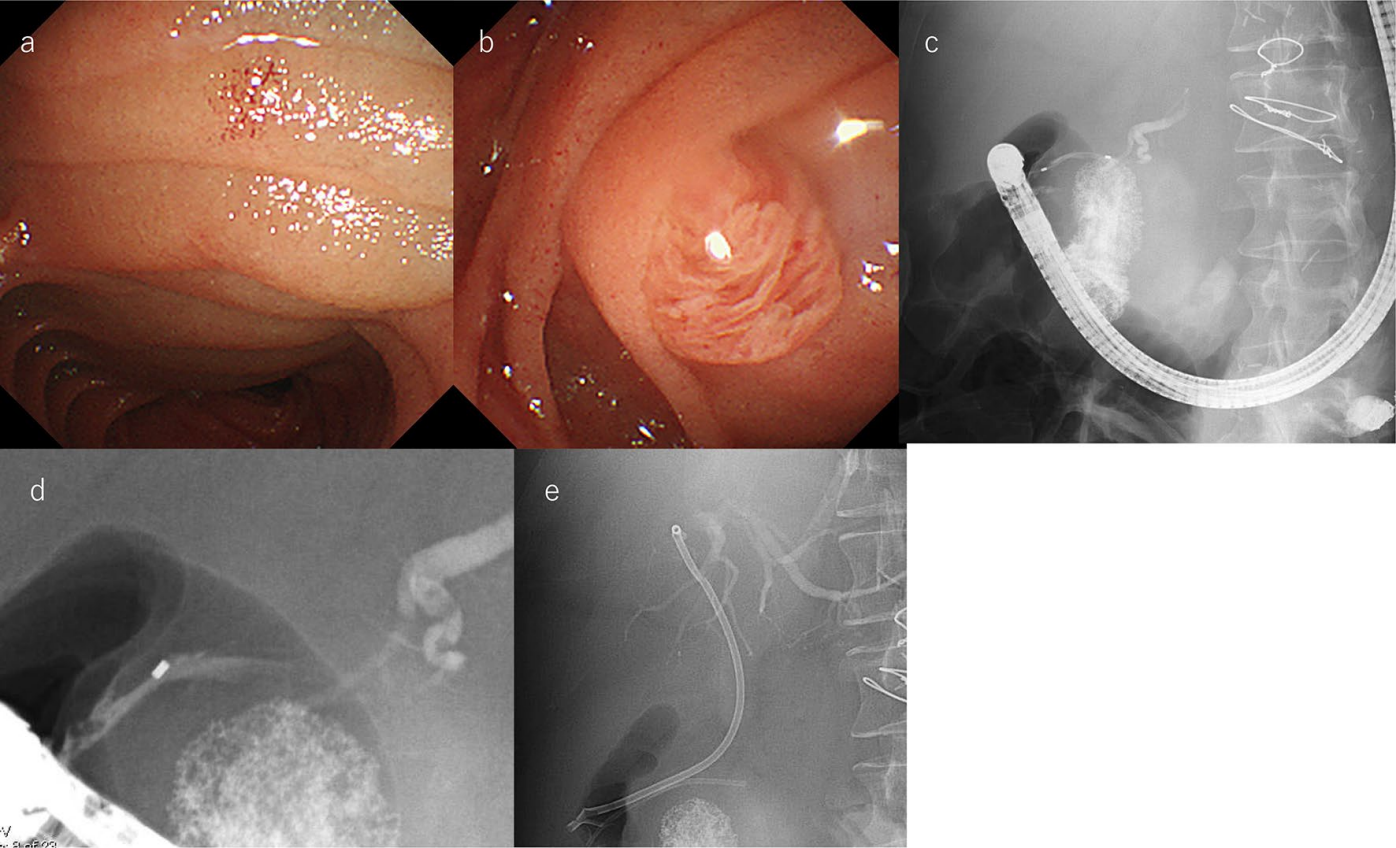
Endoscopic intervention. **a** The duodenum showed severe deformity due to compression by pseudoaneurysm, and the duodenal mucosa showed edematous changes due to inflammation. **b** The major papilla was also enlarged and displaced as a result. **c** The scope position was unstable due to the compression of a pseudoaneurysm. **d** Endoscopic retrograde pancreatography revealed compression and stenosis of the main pancreatic duct at the pancreatic head, with upstream dilation of the distal pancreatic duct. **e** Both a biliary stent and a pancreatic stent were inserted.

TAE was successfully conducted using n-butyl-2-cyanoacrylate (NBCA) and coils, effectively occluding the pseudoaneurysm (Fig. 2). The following day, the patient underwent endoscopic retrograde cholangiopancreatography (ERCP). The duodenum was severely deformed due to compression by the pseudoaneurysm, and the duodenal mucosa showed edematous changes due to inflammation (Fig. 3a). The major papilla was displaced and protruded (Fig. 3b), making the procedure challenging (Fig. 3c). Although the guidewire repeatedly entered the submucosa under wire-guided cannulation (WGC), the pancreatic and bile ducts were eventually obtained for drainage. Endoscopic retrograde pancreatography (ERP) revealed compression and stenosis of the MPD at the pancreatic head, with upstream dilation of the distal pancreatic duct (Fig. 3d). Similarly, endoscopic retrospective cholangiography (ERC) showed compression, stenosis, and angulation of the CBD. To decompress both the bile and pancreatic duct, a 5Fr × 5 cm pancreatic stent (Geenen, Cook Medical, Bloomington, IN, USA) was inserted into the MPD, while a 7Fr × 14 cm biliary stent (K-Hilar, Gadelius Medical, Tokyo, Japan) was placed in the CBD (Fig. 3e). The patient’s pancreatic and liver enzyme levels improved steadily.

**Fig. 4:**
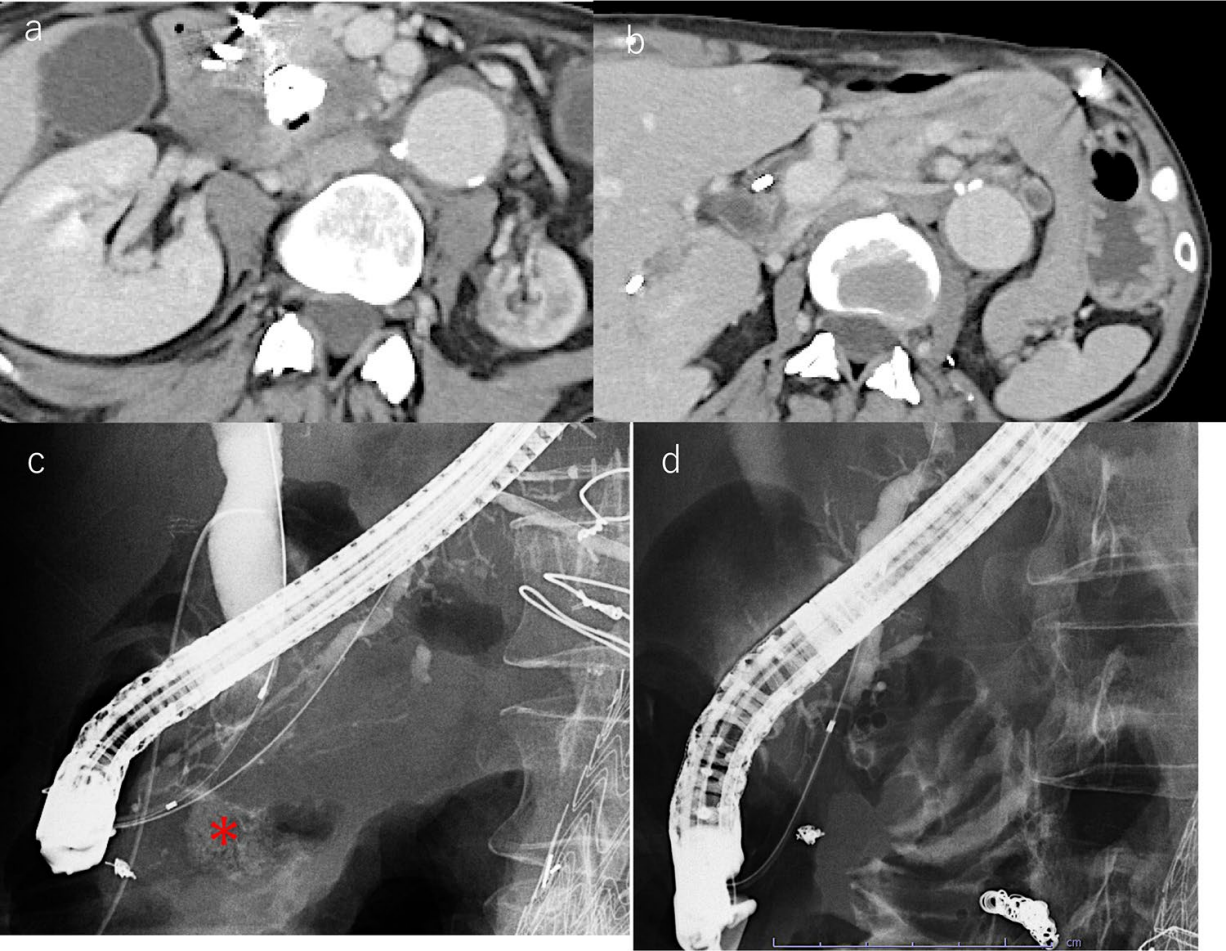
Follow-up images. **a** Contrast-enhanced CT obtained 4 months after TAE showing marked shrinkage of the pseudoaneurysm. A small amount of air is visible within the lesion, suggestive of fistula formation between the pseudoaneurysm and the duodenum. This finding improved with conservative management. **b** CT image obtained 4 months after TAE showing improvement in pancreatic swelling, indicating resolution of acute pancreatitis following pancreatic duct stent placement. **c** Four months after TAE, when contrast material was injected into the pancreatic duct, the post-TAE pseudoaneurysm (*) was opacified through the stenotic main pancreatic duct at the pancreatic head. **d** One year after TAE, no contrast material leakage was observed

Four months later, CT revealed that the pseudoaneurysm had markedly decreased in size, and a small amount of air was observed within the lesion, indicating fistula formation with the adjacent duodenum (Fig. 4a). This was managed conservatively without invasive intervention. Pancreatitis also improved following placement of a pancreatic duct stent (Fig. 4b). ERCP was performed to reassess the bile and pancreatic duct strictures, which persisted. In addition, contrast injection into the pancreatic duct revealed drainage from the stenotic MPD at the pancreatic head into the pseudoaneurysm (Fig. 4c), suggesting a fistula formation. Notably, the pseudoaneurysm showed no blood flow due to prior TAE. One year after the initial stent placement, the pseudoaneurysm was no longer opacified on pancreatography (Fig. 4d). However, because the strictures persisted, stent exchanges for the bile and pancreatic ducts were performed every 4 months. Given the risk of pseudoaneurysm formation or bleeding due to mechanical stimulation in this high-risk patient, we chose to avoid the use of larger diameter or metal stents and continued with scheduled plastic stent replacements.

## Discussion

Multiple aneurysms are a major vascular complication of LDS due to TGF-β-mediated vascular fragility [1, 2]. The average life expectancy of patients with LDS has been reported to range from 26 to 37 years [6, 7]. However, recent medical advances have improved the prognosis, with 10-year survival rates ranging from 70 to 90% [8, 9]. Early detection and treatment of ruptured aortic aneurysms and arterial dissections are crucial for prolonging the prognosis. Although aneurysms frequently occur in major arteries, pseudoaneurysms of the pancreaticoduodenal artery leading to pancreatic or bile duct obstruction are extremely rare. Pamela et al. reported the only reported case of acute pancreatitis caused by LDS [10]. However, they did not describe the treatment strategies. To the best of our knowledge, this is the first report to provide a management approach for aneurysm-induced pancreaticobiliary obstruction in LDS.

In our case, a giant pancreaticoduodenal artery pseudoaneurysm caused by LDS led to acute pancreatitis and obstructive liver dysfunction. The etiology of the pseudoaneurysm in this case warrants further consideration. Pseudoaneurysms can develop secondary to inflammation, trauma, or interventional procedures. However, our patient had no history of acute pancreatitis, abdominal trauma, intra-abdominal infections, or other inflammatory conditions. Given his known diagnosis of Loeys–Dietz syndrome and a history of multiple systemic aneurysms, we considered that the pseudoaneurysm was most likely due to LDS-associated vascular fragility. Imaging findings such as delayed contrast enhancement and partial thrombosis supported the diagnosis of a pseudoaneurysm. Nevertheless, we acknowledge the possibility that a true aneurysm may have partially ruptured and evolved into a pseudoaneurysmal configuration. As the pseudoaneurysm led to pancreaticobiliary obstruction, endoscopic drainage was necessary but carried a risk of pseudoaneurysm rupture. Therefore, TAE was performed first to mitigate the risk. Performing ERCP before embolization could have increased intra-abdominal pressure or caused direct mechanical injury, potentially leading to rupture. CT also revealed that the pseudoaneurysm compressed the duodenum, suggesting that mechanical stimulation from the endoscope insertion itself could have further increased the risk of rupture. In addition, the major papilla was protruded by the pseudoaneurysm, and the guidewire was repeatedly misdirected into the submucosal layer due to mucosal displacement during cannulation. If TAE had not been performed before ERCP, the risk of pseudoaneurysm rupture with a scope or a guidewire during ERCP would have been significantly higher. A stepwise approach involving TAE followed by ERCP was important in mitigating these risks. Although performing ERCP after TAE is a reasonable approach to minimize the risk of rupture in the presence of large pseudoaneurysms, there is no established standard strategy for such rare cases. Therefore, treatment decisions should be based on a careful evaluation of the individual anatomical and clinical context. Several previous reports have described hybrid therapy combining endoscopic intervention and IVR to treat biliary stenosis caused by aneurysms or pseudoaneurysms. Bramucci et al. reported a case of obstructive jaundice caused by celiac artery aneurysm [11]. In this case, TAE was performed prior to endoscopic drainage to prevent rupture. Similarly, El Ouardi et al. described a case of biliary obstruction secondary to a hepatic artery aneurysm, in which a hybrid approach using TAE and endoscopic treatment was successfully employed [12]. These cases, along with our experience, support the principle that under present rupture risk, IVR should be prioritized prior to endoscopic intervention. However, caution should be exercised when complicated by severe cholangitis. In such cases, immediate drainage may be preferred over IVR; however, great care must be taken to avoid rupture of the pseudoaneurysms. Furthermore, a backup system must be in place to respond immediately to rupture events. If the case is considered dangerous for endoscopic intervention, there is an alternative option to treat the cholangitis with percutaneous transhepatic biliary drainage and perform an elected endoscopic intervention after IVR. In any case, aneurysm rupture can be fatal. Therefore, it is necessary to develop a treatment plan to minimize the risk of rupture.

In our case, the endoscopic procedure was technically challenging due to severe duodenal deformation and mucosal edematous changes induced by the inflammation of the embolized pseudoaneurysm. Under these conditions, wire-guided cannulation is complicated by frequent misdirection of the guidewire into the submucosal layer. Careful fluoroscopic guidance and gentle manipulation are necessary for successful pancreatic and biliary drainage.

Four months after the first ERCP, pancreatography revealed a fistula between the MPD and pseudoaneurysm after TAE. Choi et al. reported that TAE creates a localized inflammatory response to occluded tissue necrosis, which can mimic or precipitate infection if severe [13]. This mechanism is known as postembolization syndrome (PES). Furthermore, there are various reports of inflammation in acute and chronic pancreatitis, causing fistulas with surrounding tissues, such as blood vessels and the gastrointestinal tract [14, 15]. In our case, we considered that the fistula was caused by inflammation of either the PES or acute pancreatitis. However, 8 months after the initial drainage, the fistula resolved. We attributed its resolution to pancreatic duct stent placement, which likely prevented pancreatic fluid from leaking into the fistula.

This case highlights the importance of prioritizing IVR before attempting endoscopic intervention for pseudoaneurysm-related pancreaticobiliary obstruction to prevent pseudoaneurysm rupture. Long-term stent management was necessary as strictures persisted despite the resolution of the fistula, emphasizing the need for individualized follow-up strategies.
